# Supplementary material for: Magnetically programmable surface acoustic wave filters: device concept and predictive modeling
Source: Npj Spintron. 2026 Mar 27;4(1):13. doi: 10.1038/s44306-026-00132-4 (PMC13031121; doi:10.1038/s44306-026-00132-4)
Supplement: Supplementary file 1 — Supplementary information [file 44306_2026_132_MOESM1_ESM.pdf]

Supplementary Information

Uni-Directional Model: Extension to non-linear excitations

The uni-directional model could be extended to non-linear regions of the LLG, where the exponential ansatz no longer holds, by introducing an iterative approach: Starting from a specific amplitude A_0 one calculates the starting energy of the phonon $E_{\text{Ph, 0}}$, simulates until condition $\frac{\mathrm{d}R_{\text{T}}}{\mathrm{d}t} = 0$ is reached and determines $\frac{\mathrm{d}E_{\text{Ph}}}{\mathrm{d}t}$. With this information, one can then calculate the amplitude A_1 the phonon has a small distance $\Delta l = c\Delta t$ away by making use of a first order Taylor approximation and $E_{\text{Ph}} \propto A^2$:

$$\frac{E_{\text{Ph, n+1}}}{E_{\text{Ph, 0}}} = \frac{A^2_{\text{n+1}}}{A^2_0} \tag{1}$$

$$\Rightarrow A_{\text{n+1}} = A_0\sqrt{\frac{E_{\text{Ph, n+1}}}{E_{\text{Ph, 0}}}} \tag{2}$$

$$= A_0\sqrt{\frac{E_{\text{Ph, n}} + \Delta t\frac{\mathrm{d}E_{\text{Ph, n}}}{\mathrm{d}t}}{E_{\text{Ph, 0}}}} \tag{3}$$

$$= A_0\sqrt{\frac{E_{\text{Ph, n}}}{E_{\text{Ph, 0}}} + \frac{\frac{\Delta l}{c}\frac{\mathrm{d}E_{\text{Ph, n}}}{\mathrm{d}t}}{E_{\text{Ph, 0}}}} \tag{4}$$

$$= A_0\sqrt{\frac{A^2_{\text{n}}}{A^2_0} + \frac{\Delta l}{c \cdot E_{\text{Ph, 0}}}\int_\Omega \left(\frac{\partial}{\partial t}\varepsilon\right) : C : \varepsilon_{\boldsymbol{m}}\,\mathrm{d}\boldsymbol{x}.} \tag{5}$$

Repeating this process of determining $\frac{\mathrm{d}E_{\text{Ph}}}{\mathrm{d}t}$ with the new amplitude and then readjusting it, N times, one arrives at the transmission losses ΔS_{ij} after the distance $l = N\Delta l$:

$$\Delta S_{ij}(l) = 10\log_{10}\left(\frac{P_{\text{out}}}{P_{\text{in}}}\right) \tag{6}$$

$$= 10\log_{10}\left(\frac{A^2_{\text{N}}}{A^2_0}\right). \tag{7}$$

In cases where the exponential ansatz does hold, so if $\frac{\mathrm{d}E_{\text{Ph, n}}}{\mathrm{d}t} = \beta E_{\text{Ph, n}} \forall n$ with $\beta = const.$, then this iterative algorithm is equivalent to the regular uni-directional model for infinitesimal Δl:

$$\frac{A^2_{\text{N}}}{A^2_0} = \frac{E_{\text{Ph, N}}}{E_{\text{Ph, 0}}} \tag{8}$$

$$= \frac{E_{\text{Ph, N-1}} + \Delta t\frac{\mathrm{d}E_{\text{Ph, N-1}}}{\mathrm{d}t}}{E_{\text{Ph, 0}}} \tag{9}$$

$$= \frac{E_{\text{Ph, N-1}} + \frac{\Delta l}{c}\beta E_{\text{Ph, N-1}}}{E_{\text{Ph, 0}}} \tag{10}$$

$$= \frac{\left(1 + \frac{\Delta l}{c}\beta\right) E_{\text{Ph, N-1}}}{E_{\text{Ph, 0}}} \tag{11}$$

$$= \frac{\left(1 + \frac{\Delta l}{c}\beta\right)\left(E_{\text{Ph, N-2}} + \frac{\Delta l}{c}\beta E_{\text{Ph, N-2}}\right)}{E_{\text{Ph, 0}}} \tag{12}$$

$$= \frac{\left(1 + \frac{\Delta l}{c}\beta\right)^2 E_{\text{Ph, N-2}}}{E_{\text{Ph, 0}}} \tag{13}$$

$$\vdots$$

$$= \frac{\left(1 + \frac{\Delta l}{c}\beta\right)^N E_{\text{Ph, N-N}}}{E_{\text{Ph, 0}}} \tag{14}$$

$$= \left(1 + \frac{\Delta l}{c}\beta\right)^N \tag{15}$$

$$= \left(1 + \frac{\Delta l}{c}\beta\right)^{\frac{l}{\Delta l}} \tag{16}$$

$$= \left(\left(1 + \frac{\Delta l}{c}\beta\right)^{\frac{1}{\Delta l}}\right)^l \tag{17}$$

$$\Rightarrow \lim_{\Delta l \to 0}\frac{A^2_{\text{N}}}{A^2_0} = \lim_{\Delta l \to 0}\left(\left(1 + \frac{\Delta l}{c}\beta\right)^{\frac{1}{\Delta l}}\right)^l \tag{18}$$

$$= \left(\lim_{\Delta l \to 0}\left(1 + \frac{\Delta l}{c}\beta\right)^{\frac{1}{\Delta l}}\right)^l \tag{19}$$

$$= \left(\exp\left(\frac{1}{c}\beta\right)\right)^l \tag{20}$$

$$= \exp\left(\frac{l}{c}\beta\right) \tag{21}$$

$$\Rightarrow 10\log_{10}\left(\frac{A^2_{\text{N}}}{A^2_0}\right) = 10\log_{10}\left(\exp\left(\frac{l}{c}\beta\right)\right). \tag{22}$$

Comparison of magneto-rotational to magneto-elastic effect

To quantify the expected contribution incorporating magneto-rotational coupling would have on our simulation, we will follow an argument employed in ref. [1]. To find the magneto-rotational coupling coefficients $K_{\alpha\beta}$, we first examine the energy density related to the anisotropy E_a:

$$E_a = \frac{1}{2}\mu_0 M_{\text{s}}^2 \boldsymbol{m}^T \cdot N \cdot \boldsymbol{m} - K_u(\boldsymbol{m} \cdot \boldsymbol{e}_u)^2 \tag{23}$$

In our case, $\boldsymbol{e}_u = \hat{\boldsymbol{z}}$. Using the approximation for the demag tensor of thin plates ($N_{xx} = N_{yy} = 0, N_{zz} = 1$) this simplifies to:

$$E_a = \frac{1}{2}\mu_0 M_\mathrm{s}^2 (\boldsymbol{m}\cdot\hat{\boldsymbol{z}})^2 - K_u (\boldsymbol{m}\cdot\hat{\boldsymbol{z}})^2 \tag{24}$$

$$= \left(\frac{1}{2}\mu_0 M_\mathrm{s}^2 - K_\mathrm{u}\right)(\boldsymbol{m}\cdot\hat{\boldsymbol{z}})^2 \tag{25}$$

Introducing a small rotation $\delta\hat{\boldsymbol{z}} = \omega_{xz}\hat{\boldsymbol{x}} + \omega_{yz}\hat{\boldsymbol{y}}$:

$$E_a = \left(\frac{1}{2}\mu_0 M_\mathrm{s}^2 - K_\mathrm{u}\right)(\boldsymbol{m}\cdot(\hat{\boldsymbol{z}} + \delta\hat{\boldsymbol{z}}))^2 \tag{26}$$

$$= \left(\frac{1}{2}\mu_0 M_\mathrm{s}^2 - K_\mathrm{u}\right)((\boldsymbol{m}\cdot\hat{\boldsymbol{z}})^2 + 2(\boldsymbol{m}\cdot\hat{\boldsymbol{z}})(\boldsymbol{m}\cdot\delta\hat{\boldsymbol{z}}) + (\boldsymbol{m}\cdot\delta\hat{\boldsymbol{z}})^2) \tag{27}$$

Disregarding the term quadratic in $\delta\hat{\boldsymbol{z}}$ and multiplying:

$$E_a = \left(\frac{1}{2}\mu_0 M_\mathrm{s}^2 - K_\mathrm{u}\right)(m_z^2 + 2\omega_{xz} m_x m_z + 2\omega_{yz} m_y m_z) \tag{28}$$

Comparing this to the general form of the magneto-rotational energy density $E_\mathrm{MR} = \sum_{\alpha\beta} K_{\alpha\beta}\omega_{\alpha\beta} m_\alpha m_\beta$ we find for the magneto-rotational coupling coefficients:

$$K_{xz} = K_{yz} = \left(\frac{1}{2}\mu_0 M_\mathrm{s}^2 - K_\mathrm{u}\right) = -K_{zx} = -K_{zy} \tag{29}$$

$$K_{xy} = K_{yx} = K_{xx} = K_{yy} = K_{zz} = 0 \tag{30}$$

One can also obtain the same coefficients by inserting the thin plate approximation ($N_{xx} = 0, N_{zz} = 1$) directly in the coefficients given in ref. [1]. For a Rayleigh wave: $\omega_{yz} = 0$. Keeping in mind that $K_{xz}\omega_{xz} = K_{zx}\omega_{zx}$ we can therefore write the magneto-rotational energy density as:

$$E_\mathrm{MR} = 2K_{xz}\omega_{xz} m_x m_z \tag{31}$$

The effective field is then:

$$\boldsymbol{H}^{MR} = -\frac{1}{\mu_0 M_\mathrm{s}}\frac{\delta E_\mathrm{MR}}{\delta \boldsymbol{m}} \tag{32}$$

$$= -\frac{1}{\mu_0 M_\mathrm{s}}\begin{pmatrix}2K_{xz}\omega_{xz}m_z\\ 0\\ 2K_{xz}\omega_{xz}m_x\end{pmatrix} \tag{33}$$

This is additive to the effective magneto-elastic field for a Rayleigh wave ($\varepsilon_{xy} = \varepsilon_{yz} = \varepsilon_{yy} = 0$):

$$\boldsymbol{H}^{ME} = -\frac{1}{\mu_0 M_\mathrm{s}}\begin{pmatrix}2b(\varepsilon_{xx}m_x + \varepsilon_{xz}m_z)\\ 0\\ 2b(\varepsilon_{zz}m_z + \varepsilon_{xz}m_x)\end{pmatrix} \tag{34}$$

Here we again assumed a polycrystalline material with at least cubic symmetry (where $b = -3C_{44}\lambda_s$ [2]). While ω_{xz} is on the same order of magnitude as the main strain components of the Rayleigh mode, K_{xz} is much smaller than b for both:

- The islet design: $K_{xz} \approx -35.74\,\mathrm{kJ/m^3}$ and $b \approx 16.63\,\mathrm{MJ/m^3}$.
- And the experiment in ref. [3]: $K_{xz} \approx 80.79\,\mathrm{kJ/m^3}$ and $b \approx 3.58\,\mathrm{MJ/m^3}$.

We therefore decided to disregard magneto-rotational coupling in our analysis.

[1] G. Centała and J. W. Kłos, Magneto-rotation coupling for ferromagnetic nanoelement embedded in elastic substrate, Journal of Applied Physics **137**, 10.1063/5.0271755 (2025).

[2] L. Dreher, M. Weiler, M. Pernpeintner, H. Huebl, R. Gross, M. S. Brandt, and S. T. Goennenwein, Surface acoustic wave driven ferromagnetic resonance in nickel thin films: Theory and experiment, Physical Review B - Condensed Matter and Materials Physics **86**, 10.1103/PhysRevB.86.134415 (2012).

[3] M. Küß, M. Heigl, L. Flacke, A. Hefele, A. Hörner, M. Weiler, M. Albrecht, and A. Wixforth, Symmetry of the magnetoelastic interaction of rayleigh and shear horizontal magnetoacoustic waves in nickel thin films on litao3, Physical Review Applied **15**, 10.1103/PhysRevApplied.15.034046 (2021).
